# FimAsartaN proTeinuriA SusTaIned reduCtion in comparison with losartan in diabetic chronic kidney disease (FANTASTIC): study protocol for randomized controlled trial

**DOI:** 10.1186/s13063-017-2375-8

**Published:** 2017-12-29

**Authors:** Jang-Young Kim, Jung-Woo Son, Sungha Park, Tea-Hyun Yoo, Yong-Jin Kim, Dong-Ryeol Ryu, Ho Jun Chin

**Affiliations:** 1 Division of Cardiology, Department of Internal Medicine, Yonsei University Wonju College of Medicine, Wonju, Republic of Korea; 2 Divison of Cardiology, Severance Cardiovascular Hospital and Cardiovascular Research Institute, Yonsei University College of Medicine, Seoul, Republic of Korea; 3 Department of Internal Medicine, Yonsei University College of Medicine, Seoul, Republic of Korea; 4 Cardiovascular Center, Seoul National University Hospital, Seoul, Republic of Korea; 5 Department of Internal Medicine, School of Medicine, Ewha Womans University, 1071, Anyangcheon-ro, Yangcheon-gu, Seoul 158-710 Republic of Korea; 6 Division of nephrology, Department of internal medicine, Seoul National University Bundang Hospital, Seongnam, Gyeonggi-do Republic of Korea

**Keywords:** Proteinuria, Chronic kidney disease, Hypertension, Diabetes mellitus

## Abstract

**Background:**

Fimasartan is the ninth angiotensin receptor blocker to be developed. However, it has not yet been evaluated for reno-protective effects in hypertensive diabetic chronic kidney disease (CKD). The target blood pressure (BP) for hypertensive diabetic CKD is also a controversial topic. This trial was designed to assess the reno-protective effects of fimasartan compared to those of losartan as a primary outcome. This study also compares the two drugs with regard to cardiovascular and renal outcomes in accordance with target systolic BP (SBP) (as secondary outcomes).

**Methods:**

This study is a prospective, phase III, randomized, double-blind, active-controlled, non-inferiority, four-parallel group, dose-titration, multicenter trial. We recruit patients with hypertensive diabetic CKD with overt proteinuria. Participants will be randomized into four groups (1:1:1:1): fimasartan standard SBP control (SBP < 140 mmHg); fimasartan strict SBP control (SBP < 130 mmHg); losartan standard SBP control; and losartan strict SBP control. After 24 weeks, all individuals are treated with fimasartan for an additional 120 weeks in an open-label design, maintaining their assigned SBP control groups as randomized. The primary endpoint is the rate of change in proteinuria, which is assessed using the spot urine albumin–creatinine ratio at 24 weeks. The secondary endpoints are the cardiovascular and renal outcomes at 144 weeks compared between the strict SBP and standard SBP control groups.

**Discussion:**

The FANTASTIC is a clinical study to provide: (1) the reno-protective effect of fimasartan; and (2) the target BP to reduce adverse outcomes in hypertensive diabetic CKD with overt proteinuria.

**Trial registration:**

Clinicaltrials.gov, NCT02620306. Registered on 1 December 2015.

**Electronic supplementary material:**

The online version of this article (doi:10.1186/s13063-017-2375-8) contains supplementary material, which is available to authorized users.

## Background

Hypertension affects approximately 40% of adults worldwide [1] and about two-thirds of patients with diabetes have concomitant hypertension [2]. Both hypertension and diabetes have a variety of vascular complications, including macrovascular and microvascular effects. The macrovascular complications of hypertension and diabetes include coronary artery disease, stroke, and peripheral vascular disease. The microvascular complications include retinopathy, nephropathy, and neuropathy. Hypertension and diabetes also increase the risk of developing new-onset chronic kidney disease (CKD) [3]. The progression of CKD is accompanied by microvascular complications. CKD increases a patient’s risk of stroke, coronary artery disease, and all-cause mortality. The annual mortality rate of those patients who progress to require dialysis is about 10–20% [4, 5].

Given the association between CKD and cardiovascular disease, it is important to suppress CKD progression in patients with both hypertension and diabetes. The degree of baseline proteinuria is correlated to the future progression of CKD [6–8]. High albuminuria was shown to be associated with increased risk of myocardial infarction, stroke, first hospitalization for heart failure, unstable angina, coronary or peripheral revascularization, and cardiovascular death [9]. Recent evidence has shown that reducing proteinuria can prevent the progression of CKD. Angiotensin II receptor blockers (ARB) have also been shown to reduce CKD progression of CKD [10–14]. Most hypertension guidelines recommend that angiotensin converting enzyme inhibitors (ACEI) or ARBs be used in patients with hypertensive diabetes [15–17]. There are few studies that directly compare the renal efficacy of two ARBs in hypertensive diabetes. In one comparison study of hypertensive type 2 diabetic patients with overt nephropathy, telmisartan were found to have similar renal efficacy to valsartan [18]. In another comparison study, telmisartan was found to be superior to losartan at reducing the geometric mean urinary protein–creatinine ratio without a change in blood pressure (BP), dietary sodium, or estimated glomerular filtration rate (eGFR) [19]. Therefore, although ARBs have a reno-protective class effect, there are relative differences in each drug’s efficacy.

Fimasartan was the ninth ARB approved for the treatment of hypertension by the Korean Ministry of Food and Drug Safety in 2010; it entered the market in 2011 [20]. Its chemical characteristics are as follows: 2-n-butyl-5-dimethylaminothiocarbonyl methyl-6-methyl-3-([2’-(1H-tetrazol-5-yl) biphenyl-4-yl] methyl) pyrimidin-4(3H)-one); molecular formula, C27H31N7OS; molecular weight, 501.65; formally known as BR-A-657. Despite the proven antihypertensive effect, however, the renal efficacy and safety of fimasartan in hypertensive diabetic CKD have not been studied or compared to those of other ARBs. Therefore, this randomized multicenter clinical trial compares the renal efficacy and safety of fimasartan to those of losartan, which has already been shown to have a renal protective effect [12].

The target BP in diabetic CKD remains controversial. There are inconsistencies in the current guidelines regarding target BP for patients with diabetic CKD from KDIGO (Kidney Disease Improving Global Outcomes), ESC (European Society of Cardiology), and JNC (Joint National Committee) 8 [15, 16, 21]. The current KDIGO guideline recommends that the target goal is a systolic BP (SBP) < 130 mmHg and diastolic BP (DBP) < 80 mmHg in diabetic CKD, with a urine albumin excretion > 30 mg/day. [21] The JNC 8 panel recommends that hypertensive adults with diabetic or non-diabetic CKD keep their BP < 140/90 mmHg. [15]

Thus, the purpose of the present study, FimAsartaN proTeinuriA SusTaIned reduCtion in comparison with losartan in diabetic chronic kidney disease (FANTASTIC) trial, is to investigate the reno-protective effect of fimasartan in comparison with losartan in patients within a diabetic CKD group and we also aim to evaluate the long-term effect of strict SBP control on renal and cardiovascular outcomes in comparison with standard SBP control.

## Methods

### Study design

The FANTASTIC study is a randomized, multi-centered, double-blind, four-parallel group, dose-titration, phase III study. This study was designed to compare the efficacy of fimasartan (study group) and losartan (control group) with regard to the rate of change in proteinuria. It also compares the adverse outcomes between strict BP control and standard BP control in patients with hypertensive diabetic CKD with overt proteinuria. Forty clinical research centers (19 nephrology, 14 cardiology, and seven endocrinology) participated in this trial and all research centers are comprised of the tertiary university hospital. This study is sponsored by an investigator-initiated grant by Boryung Pharmaceutical Co., Ltd. It was registered at http://www.clinicaltrials.gov/ before participant recruitment (NCT02620306). All participants will provide written informed consent based on documents approved by each university Institutional Review Board.

### Study eligibility

#### Inclusion and exclusion criteria

Patients are screened for the following four required conditions: high BP; CKD; overt proteinuria (or macroalbuminuria); and type 2 diabetes mellitus. The detailed inclusion criteria are described in Table 1. The exclusion criteria are described in Table 2. Finally, after learning about the study, patients voluntarily provided written consent to participate.

**Table 1:** Inclusion criteria

Age: 19–70 years
Screening visit (Visit 1)
Hypertension in treatment-naïve patients
140 mmHg ≤ SBP < 180 mmHg and DBP < 110 mmHg
Hypertension with treatment, including ACEI/ARB
130 mmHg ≤ SBP < 180 mmHg and DBP < 110 mmHg
eGFR ≥ 30 mL/min/1.73 m^2^ (by MDRD) within the past six months
Urine protein (within the past six months)
ACR ≥ 300 mg/g (or mg/day)
or protein–creatinine ratio ≥ 500 mg/g (or mg/day)
Type 2 diabetes mellitus (diagnosed > 3 months prior) on medications with
no changes in medication regimen/dose within the last three months
Baseline visit (Visit 3)
Hypertension
140 mmHg ≤ SBP < 180 mmHg and DBP < 110 mmHg
eGFR ≥ 30 mL/min/1.73 m^2^ (by MDRD)
Urine protein: ACR ≥ 300 mg/g in spot urine
Type 2 diabetes without regimen change during placebo run-in period

*SBP* systolic blood pressure, *DBP* diastolic blood pressure, *ACEI* angiotensin converting enzyme inhibitor, *ARB* angiotensin receptor blocker, *ACR* albumin–creatinine ratio, *eGFR* estimated glomerular filtration rate, *MDRD* Modification of Diet in Renal Disease

**Table 2:** Exclusion criteria

Secondary or malignant hypertension
Inter-arm BP difference: SBP > 20 mmHg or DBP > 10 mmHg
Symptomatic orthostatic hypotension
Cardiovascular history
MI, congestive heart failure (NYHA class III or IV), CABG, PTCA, or angina < 3 months before enrollment
Severe cerebrovascular disease (stroke, cerebral infarction, or cerebral hemorrhage)
Arrhythmia (significant ventricular tachycardia, atrial fibrillation, or atrial flutter)
Significant valvular disease: aortic stenosis or mitral stenosis
Hypertrophic cardiomyopathy
Type 1 DM or HbA1c > 9% at screening
Significant renal or hepatic disease
Dialysis, cirrhosis, biliary tract obstruction, cholestasis, or liver failure
eGFR (MDRD) < 30 mL/min/1.73 m2, hyperkalemia (>5.5 mmol/L) or hypokalemia (<3.5 mmol/L)
AST or ALT > 3 times the upper limit of normal
Any chronic inflammatory disease requiring chronic anti-inflammatory treatment (including rheumatoid arthritis, systemic lupus erythematosus, or connective tissue disease)
Moderate or malignant retinopathy < 6 months before enrollment (e.g. moderate or severe non-proliferative diabetic retinopathy and proliferative diabetic retinopathy)
Surgical or medical disease that may affect drug absorption, distribution, metabolism, or excretion
Cancer history with current treatment or treatment within five years
Pregnancy, childbearing potential without adequate contraception, or breast-feeding
Previous hypersensitivity reaction to renin-angiotensin system inhibitors
Alcohol or drug abuse within the previous two years
Poor compliance during placebo run-in period: < 70%

*SBP* systolic blood pressure, *DBP* diastolic blood pressure, *MI* myocardial infarction, *NYHA* New York Heart Association, *CABG* coronary artery bypass graft, *PTCA* percutaneous coronary angioplasty, *DM* diabetes mellitus, *eGFR* estimated glomerular filtration rate, *MDRD* Modification of Diet in Renal Disease, *AST* aspartate transaminase, *ALT* alanine transaminase

### Screening

Included individuals go through a single-blind placebo run-in period for at least four weeks. During this period, any treatment with ACEI/ARBs for hypertension will be discontinued. However, other antihypertensive drugs are administered without changing the regimens or doses. During the placebo run-in period, the participant is instructed to measure his/her own BP with a provided oscillometric automatic device more than once a day. Individuals are instructed to visit the study site in the event of SBP ≥ 180 mmHg or onset of any symptoms of suspected hypertension (e.g. headache, dyspnea, chest discomfort, vomiting, and neurologic symptoms). In this situation, additional antihypertensive drugs (other than ACEIs/ARBs) are introduced or adjustments were made to the doses of the patient’s other medications as appropriate. After the placebo run-in period is completed for at least four weeks, tests for eligibility assessment are performed at the visit before baseline (Visit 2).

#### Randomization

**Fig. 1:**
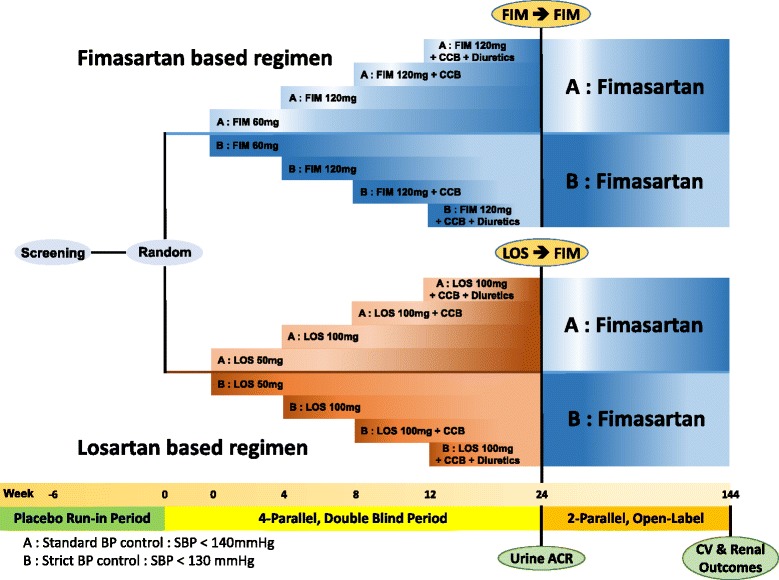
*Flow chart* of FANTASTIC study. The FANTASTIC study consists of two phases: four-parallel double-blind period to investigate the reno-protective effect of fimasartan and two-parallel open-label period for prognosis in accordance with target BP. **a** Standard BP control group (systolic BP < 140 mmHg). **b** Strict BP control group (systolic BP < 130 mmHg). FIM fimasartan, LOS losartan, CCB calcium channel blocker, ACR albumin–creatinine ratio, CV cardiovascular, SBP systolic blood pressure

The individuals are finally selected at baseline (Visit 3). An independent group not involved with study implementation creates a randomization schedule for study drug labelling and randomization is stratified by study site. Eligible participants are randomized into one of the following four groups in a 1:1:1:1 ratio using the interactive web-based randomization system, Fimasartan group A, Fimasartan group B, Losartan group A, and Losartan group B. Group A is a standard BP control group (SBP < 140 mmHg), while Group B is a strict BP control group (SBP < 130 mmHg). The randomized participants are treated with the investigational product (fimasartan or losartan) corresponding to each treatment group for 24 weeks on double-blind (physician and patient). After that, all individuals take fimasartan for an additional 120 weeks in an open-label study conducted with two groups (in accordance with BP control). The eligible participants provide written consent for participation in the open-label study and clinical study (Fig. 1).

**Fig. 2:**
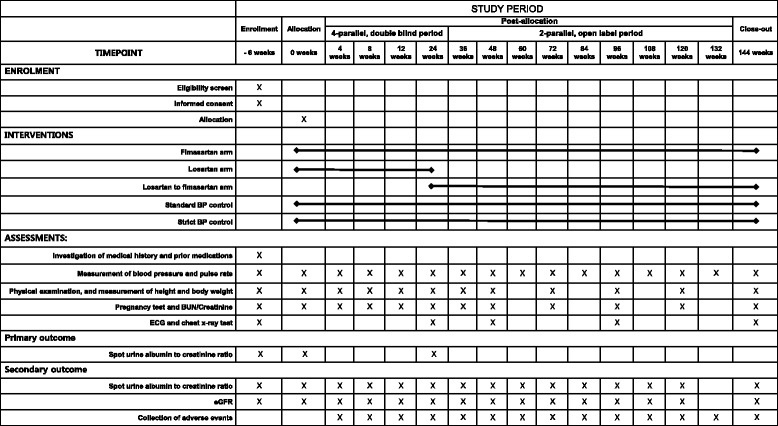
Standard Protocol Items: Recommendations for Interventional Trials (SPIRIT) figure. BP blood pressure, BUN blood urea nitrogen, ECG electrocardiography, eGFR estimated glomerular filtration rate

### Methods of dose administration

Either the study drug (fimasartan 60 mg/day) or control drug (losartan 50 mg/day) is administered at the time of randomization and maintained during the double-blind study period. The dose is titrated if the patient BP dose not meets the targeted level at each visit after Visit 4. The dose titrations are made at the discretion of the investigator based on the measured SBP. Fimasartan 60 mg or losartan 50 mg are uptitrated to fimasartan 120 mg or losartan 100 mg, respectively. At subsequent visits, additional antihypertensive therapy is added, excluding ACEIs or ARBs, in the following order: dihydropyridine calcium channel blockers; diuretics; β-blockers; α-blockers; and direct vasodilators (see Fig. 1). When additional antihypertensive drugs are administered, the dose is increased one level at a time. If the additional medication is still needed before the next visit because of continued uncontrolled BP, the dose can be titrated to the next level by making an unscheduled visit. This decision is made by the investigator. The investigator can also change the order of additional drugs administered in consideration of any antihypertensive drug that the subject have already taken. The drug dose can also be decreased in order to satisfy the BP control criteria or in the case of adverse events. However, the doses of other antihypertensive drugs (excluding fimasartan/losartan) are the first to be decreased when doses are adjusted. The doses of fimasartan and losartan are not changed if possible. Any individuals who cannot tolerate the BP control process does not continue the clinical study.

### Follow-up

The participants visit the hospital a total of seven times during the study: the screening visit; visit before baseline; baseline visit; visit 4 (four weeks); visit 5 (eight weeks); visit 6 (12 weeks); and visit 7 (24 weeks). Scheduled tests and evaluations are performed at each visit. The BP control is single-blinded in order to allow identification of the strict BP control and standard BP control groups, because the investigator should check the individual’s target BP during each follow-up. After visit 7, a two-parallel study is performed to confirm the efficacy and safety of the drug in the standard and strict BP control groups. The individuals participate in visits every 12 weeks (36, 48, 60, 72, 84, 96, 108, 120, 132, and 144 weeks) for efficacy and safety assessment (Fig. 2). All of the participants take fimasartan during the open-label study period. The individuals taking losartan during the double-blind study period will be change their drug to the equivalent dose of fimasartan. If the targeted BP cannot be reached, the dose titration method will be used in the double-blind study period (see Fig. 1).

### Blood pressure and biochemical measurements

The reported BP is the mean BP from three measurements on the selected arm. Proteinuria is measured from the spot urine albumin–creatinine ratio (ACR) based on the first urine in the morning. The eGFR is calculated using the modified Modification of Diet in Renal Disease (MDRD) equation [22].

### Primary and secondary endpoints

The primary endpoint is the rate of change in proteinuria in the fimasartan group and the losartan group from baseline to 24 weeks.

The major secondary endpoints are cardiovascular composite endpoints and renal composite endpoints. The cardiovascular composite endpoints are defined as the time to the first occurrence of any of the following (in accordance with the BP control criteria): myocardial infarction; stroke; hospitalization due to heart failure; unstable angina; coronary revascularization; peripheral revascularization; or all-cause mortality. The renal composite endpoints include any of the following (in accordance with the BP control criteria): the time to serum creatinine concentration doubling from baseline; the time to progression to end stage renal disease (ESRD) (as confirmed by the initiation of long-term dialysis or renal transplantation); and all-cause mortality.

### Other secondary endpoints

The other secondary endpoints are summarized as follows: (1) the rate of change in proteinuria in the fimasartan and losartan groups from baseline to weeks 4, 8, and 12; (2) the change in proteinuria in the fimasartan and losartan groups from baseline to weeks 4, 8, and 12; (3) the rate and amount of change in eGFR in the fimasartan and losartan groups from baseline to weeks 4, 8, 12, and 24; (4) the proportion of individuals who developed microalbuminuria (urine ACR < 300 mg/g) in the fimasartan and losartan groups at weeks 4, 8, 12, and 24; (5) the proportion of individuals with ≥ 30% decrease in albuminuria in the fimasartan and losartan groups from baseline to weeks 4, 8, 12, and 24; (6) the proportion of participants with ≥ 50% decrease in eGFR in the fimasartan and losartan groups from baseline to weeks 4, 8, 12, and 24: (7) the rate and amount of change in albuminuria from baseline to weeks 4, 8, 12, 24, 36, 48, 72, 96, 120, and 144 in accordance with the BP control criteria; (8) the rate and amount of change in eGFR from baseline to weeks 4, 8, 12, 24, 36, 48, 72, 96, 120, and 144 in accordance with the BP control criteria; (9) the proportion of individuals with a change in microalbuminuria (urine ACR < 300 mg/g) at weeks 4, 8, 12, 24, 36, 48, 72, 96, 120, and 144 in accordance with the BP control criteria; (10) the proportion of participants with ≥ 30% decrease in albuminuria from baseline to weeks 4, 8, 12, 24, 36, 48, 72, 96, 120, and 144 in accordance with the BP control criteria; and (11) the proportion of individuals with ≥ 50% decrease in eGFR from baseline to weeks 4, 8, 12, 24, 36, 48, 72, 96, 120, and 144 in accordance with the BP control criteria.

### Safety assessment

The safety assessments include laboratory tests (hematology/blood chemistry, urinalysis), electrocardiogram, chest X-ray, physical examination, orthostatic hypotension assessment, and changes in body weight and pulse rate. At least once after randomization, any adverse events that occurred after administration of the investigational product are assessed. All adverse events are summarized and presented by treatment group with respect to severity, relationship to the investigational product, and outcome.

### Data collection and management

The electronic case report form (eCRF) is developed by supervision of Boryung pharm’s data management (DM) team before first enrollment. Data entry will be performed by the investigators and clinical research coordinators at the participating sites using a web-based database: Medidata Rave™ (Medidata Solutions, Inc., http://www.mdsol.com). Rave™ is a commercial system designed to capture, manage, and report clinical research data. And Medidata Rave™ supports electronic record and electronic signature requirements, audit trail including US 21 CRF part 11. Through this system, each participating site is assigned a unique code, as identified by the study team. All staff is trained in using eCRF in advance and then is given each EDC (Electronic Data Capture)’s role. The DM team also provided at each site on the data entry and data monitoring guide book. All participating sites will use the same CRFs. If responses to the initial inclusion and exclusion criteria provided by the individual performing the data entry fulfill study criteria, the system will dynamically generate randomization and data validation process. All DM tasks are performed by Data Management Plan (DMP). We designed the eCRF and set up all DM process according the protocol and DMP documents. The DMP include all staff’s role and responsibility, each step’s definition and process, data backup and transfer, etc. Auto and manual data queries are generated by CRA (Clinical Research Associates) or a DM person and will be resolved by CRCs (Clinical Reseach Coordinators) and investigators. Through this iterative process, we will make the data clean and finally perform database lock. All database backup of eCRF will be done in real time by Medidata’s Houston data center and Boryung pharm’s database system.

### Statistics

In order to achieve a statistical power of 80%, the sample size assumed a non-inferiority margin of 18% (based on two-sided t test and a significance level of 0.025). This is based on the rate of change in proteinuria from baseline (Week 0) to week 24 of both losartan and fimasartan groups with a standard deviation of 62% [10, 12–14, 23]. Based on the assumption of a drop-out rate of 20%, a total of 468 patients are needed for randomization (with 234 patients per treatment arm).

The primary efficacy assessment finds that the fimasartan group is not inferior to the losartan group if the lower limit of a 95% two-sided confidence interval for the difference in the rate of change in proteinuria at week 24 between the fimasartan and losartan groups (control group - study group) is > − 18%. This is defined as the non-inferiority margin.

The major secondary efficacy assessment is determined by the incidence of combined cardiovascular and renal composite endpoints and is assessed using Kaplan–Meier curves, according with target BP at 144 weeks. The median survival time and confidence interval for the median survival time are presented. The log-rank test is used to compare the groups. Other secondary efficacy assessments are determined using the following methods. For continuous variables, descriptive statistics (number of individuals, mean, standard deviation, median, minimum, and maximum) for each time point are provided according to treatment group or the BP control criteria. The t-test is used to compare the inter-group difference in the rate of change. The inter-group difference in the amount of change is tested using the analysis of covariance, with the baseline values as covariates and the treatment groups as factors. The least squared mean and standard error are provided for each group. Frequencies and ratios are provided for categorical variables such as the proportion of individuals. For categorical variables, the Chi-square test, Fisher’s exact test, or Cochran–Mantel–Haenszel test is performed.

The safety analysis is based on adverse events, laboratory abnormalities, electrocardiography, chest X-rays, orthostatic hypotension assessments, and abnormal changes in body weight and pulse. Appropriate statistical analysis methods are used to compare groups with regard to differences in the rate of adverse events, abnormal laboratory values, or other parameters. Either the Chi-square test or Fisher’s exact test is used to assess differences in the incidence of adverse events between the groups. Continuous data, including laboratory values (hematology/blood chemistry, urinalysis) and vital signs (pulse rate, body weight, etc.), are presented in the form of descriptive statistics (mean, standard deviation, minimum, maximum, and median) by group and visit. Either a paired t-test or the Wilcoxon signed-rank test is used to test the intra-group difference in the continuous data between baseline and after drug administration. In addition, the two-sample t-test and Wilcoxon rank sum test is performed to test inter-group differences (in continuous variables) from baseline to after drug administration. Among all of the laboratory values, categorical data are divided into normal and abnormal results. The inter-group differences are tested using the Chi-square test or Fisher’s exact test. The McNemar’s test is used to compare differences in the categorical data between baseline and after drug administration.

## Discussion

This study compares the changes in proteinuria between fimasartan and losartan groups in patients with hypertensive diabetic CKD with overt proteinuria. It also compares the differences in cardiovascular and renal outcomes in accordance with the target SBP goal.

The majority of hypertensive guidelines recommend treating patients with diabetes or CKD with ACEIs or ARBs because of the reno-protective effect of RAAS (Renin Agiogtensin Aldosterone System) blockade [15–17]. However, there are few studies that directly compare the reno-protective effect of different ARB agents in these patients. In the VIVALDI study, telmisartan 80 mg and valsartan 160 mg had similar efficacy with regard to changes in 24-h proteinuria [18]. In contrast, in the AMADEO study, telmisartan 80 mg was superior to losartan 100 mg with regard to the change in the spot urine protein–creatinine ratio [19]. All of the patients included in the above two studies had diabetic CKD with overt proteinuria and had SBP > 130 mmHg or DBP > 80 mmHg. Telmisartan’s superiority over losartan may be a result of its longer half-life, higher lipophilicity, and stronger binding affinity for the angiotensin II type 1 receptor compared to those of losartan [19].

The chemical composition of fimasartan includes a bioisosteric replacement of the imidazole part of losartan with pyrimidin-4(3H)-one [24]. Fimasartan has theoretically similar lipophilicity and half-life to those of losartan [25]. However, fimasartan has higher potency and stronger efficacy than does losartan [26]. Losartan has already been shown to have a reno-protective effect, characterized by reduced proteinuria and a significant delay in the progression of nephropathy in patients with diabetic nephropathy. These reno-protective effects were also associated with a reduction in albuminuria in the RENNAL study [6]. The present study was designed as a non-inferiority trial to compare losartan and fimasartan with regard to the rate of change in albuminuria and to confirm the reno-protective effect of fimasartan.

The target BP level in diabetic CKD is controversial. Previous studies, including the MDRD and African American Study of Kidney Disease and Hypertension (AASK), compared standard BP control with intensive BP control in CKD. The MDRD study included patients with non-diabetic CKD stage 3 or 4 and employed either standard target BP control (of ≤ 140/90 mmHg with mean arterial pressure (MAP) ≤ 107 mmHg) or intensive BP control (of 125/75 mmHg with MAP ≤ 92 mmHg). There was no difference in the progression of CKD between the standard and intensive BP control groups [27]. However, in post hoc analysis of the MDRD study, intensive BP control was beneficial in the progression of CKD in accordance with the degree of proteinuria. Patients with proteinuria of 0.25–1.0 g/d were advised to aim for a target BP of approximately < 130/80 mmHg [8].

AASK consisted of CKD stage 3 (GFR of 20–65 mL/min/1.73 m^2^) in African Americans with hypertension. The standard target BP was ≤ 140/90 mmHg (MAP ≤ 107 mmHg), and intensive target BP was 125/75 mmHg (MAP ≤ 92 mmHg). There was no difference in the progression of CKD between the standard and intensive BP control groups [28]. However, the long-term AASK data demonstrated that the intensive BP control group with proteinuria (urine protein–creatinine ratio > 0.22) had a lower incidence of ESRD than did the normal BP control group [29]. These finding suggest that it is necessary to adjust the target BP in CKD patients depending on the degree of proteinuria.

The KDIGO guideline recommends that the target BP goal in patients with diabetic CKD with urine albumin excretion > 30 mg/day is a SBP < 130 mmHg and DBP < 80 mmHg [21]. The ESC guidelines recommend target goals of SBP < 140 mmHg in CKD or non-CKD, SBP < 130 mmHg when overt proteinuria is present, and DBP < 85 mmHg in diabetes [16]. However, in the Action to Control Cardiovascular Risk in Diabetes (ACCORD) trial, patients treated with intensive BP control (SBP < 120 mmHg) and those with eGFR < 30 mL/min/1.73 m^2^ experienced more hypotension than did those with standard BP control. Intensive BP control also did not reduce the cardiovascular outcomes [30]. This result is reflected in JNC 8. Panels of JNC 8 recommend the same threshold for hypertensive adults with diabetic or non-diabetic CKD at < 140/90 mmHg [15].

The recent SPRINT (the Systolic Blood Pressure Intervention Trial) study found that, among patients at high risk for cardiovascular events, intensive BP control (SBP < 120 mmHg) resulted in lower rates of cardiovascular events than did standard BP control (SBP < 140 mmHg). Approximately 28% of all patients with CKD and a GFR between 20 and < 60 mL/min/1.73 m^2^ in SPRINT (excluding those with diabetes and overt proteinuria) experienced no benefit with regard to renal outcomes [31]. Although there was a benefit in cardiovascular outcomes in CKD, such a result cannot be generalized to all CKD patients because patients with diabetes and proteinuria were excluded. Strict BP in this study was defined as SBP < 130 mmHg. This value was higher than the intensive control parameter employed in the ACCORD study (SBP < 120 mmHg), but was the same as that used in the ESC and KDIGO studies (SBP < 130 mmHg). This study addresses the question of target BP in patients with hypertensive diabetic CKD with overt proteinuria. This was a randomized, double-blind, active-controlled, non-inferiority, four-parallel group, dose-titration, multicenter study that evaluates the efficacy and safety of fimasartan vs losartan in patients with hypertensive diabetic CKD. It is expected to confirm whether there is the reno-protective effect of fimasartan. It also addresses the cardiovascular and renal outcomes of fimasartan in accordance with target BP as a strategy to reduce proteinuria and adverse outcomes in patients with hypertensive diabetic CKD with overt proteinuria

### Trial status

Recruitment began in November 2015 and 171 patients (314 patients in screening) were randomized to October 2017. The recruitment is an ongoing process.

## Additional file

Additional file 1: SPIRIT 2013 checklist: recommended items to address in a clinical trial protocol and related documents. (DOC 102 kb)

## Abbreviations

AASK: African American Study of Kidney Disease and Hypertension
ACEI: Angiotensin converting enzyme inhibitors
ACR: Albumin–creatinine ratio
ARB: Angiotensin II receptor blocker
BP: Blood pressure
CKD: Chronic kidney disease
DBP: Diastolic blood pressure
eGFR: Estimated glomerular filtration rate
ESC: European Society of Cardiology
ESRD: End stage renal disease
JNC 8: Joint National Committee 8
KDIGO: Kidney Disease Improving Global Outcomes
MDRD: Modification of Diet in Renal Disease
SBP: Systolic blood pressure
SPRINT: Systolic Blood Pressure Intervention Trial

## References

[1] World Health Organization (2010). World health statistics 2010.

[2] Centers for Disease Control and Prevention (2011). National Diabetes Fact Sheet, 2011.

[3] Fox CS, Larson MG, Leip EP, Culleton B, Wilson PW, Levy D (2004). Predictors of new-onset kidney disease in a community-based population. JAMA.

[4] Morbidity & Mortality. Am J Kidney Dis. 2010;55(1, Supplement 1):S83–94.

[5] Hirakata H, Nitta K, Inaba M, Shoji T, Fujii H, Kobayashi S (2012). Japanese Society for Dialysis Therapy guidelines for management of cardiovascular diseases in patients on chronic hemodialysis. Ther Apher Dial.

[6] De Zeeuw D, Remuzzi G, Parving H-H, Keane WF, Zhang Z, Shahinfar S (2004). Proteinuria, a target for renoprotection in patients with type 2 diabetic nephropathy: lessons from RENAAL. Kidney Int.

[7] Breyer JA, Bain RP, Evans JK, Nahman NS, Lewis EJ, Cooper M (1996). Predictors of the progression of renal insufficiency in patients with insulin-dependent diabetes and overt diabetic nephropathy. Kidney Int.

[8] Peterson JC, Adler S, Burkart JM, Greene T, Hebert LA, Hunsicker LG (1995). Blood pressure control, proteinuria, and the progression of renal disease: the Modification of Diet in Renal Disease Study. Ann Intern Med.

[9] Gerstein HC, Mann JF, Yi Q, Zinman B, Dinneen SF, Hoogwerf B (2001). Albuminuria and risk of cardiovascular events, death, and heart failure in diabetic and nondiabetic individuals. JAMA.

[10] Viberti G, Wheeldon NM (2002). MicroAlbuminuria Reduction with VALsartan (MARVAL) Study Investigators. Microalbuminuria reduction with valsartan in patients with type 2 diabetes mellitus a blood pressure–independent effect. Circulation.

[11] Wachtell K, Ibsen H, Olsen MH, Borch-Johnsen K, Lindholm LH, Mogensen CE (2003). Albuminuria and cardiovascular risk in hypertensive patients with left ventricular hypertrophy: the LIFE study. Ann Intern Med.

[12] Brenner BM, Cooper ME, de Zeeuw D, Keane WF, Mitch WE, Parving H-H (2001). Effects of losartan on renal and cardiovascular outcomes in patients with type 2 diabetes and nephropathy. N Engl J Med.

[13] Lewis EJ, Hunsicker LG, Clarke WR, Berl T, Pohl MA, Lewis JB (2001). Renoprotective effect of the angiotensin-receptor antagonist irbesartan in patients with nephropathy due to type 2 diabetes. N Engl J Med.

[14] Parving H-H, Lehnert H, Bröchner-Mortensen J, Gomis R, Andersen S, Arner P (2001). The effect of irbesartan on the development of diabetic nephropathy in patients with type 2 diabetes. N Engl J Med.

[15] James PA, Oparil S, Carter BL, Cushman WC, Dennison-Himmelfarb C, Handler J (2014). 2014 evidence-based guideline for the management of high blood pressure in adults: report from the panel members appointed to the Eighth Joint National Committee (JNC 8). JAMA.

[16] Mancia G, Fagard R, Narkiewicz K, Redon J, Zanchetti A, Bohm M (2013). 2013 ESH/ESC Guidelines for the management of arterial hypertension: the Task Force for the Management of Arterial Hypertension of the European Society of Hypertension (ESH) and of the European Society of Cardiology (ESC). Eur Heart J..

[17] Weber MA, Schiffrin EL, White WB, Mann S, Lindholm LH, Kenerson JG (2014). Clinical practice guidelines for the management of hypertension in the community. J Clin Hypertension.

[18] Galle J, Schwedhelm E, Pinnetti S, Böger RH, Wanner C (2008). Antiproteinuric effects of angiotensin receptor blockers: telmisartan versus valsartan in hypertensive patients with type 2 diabetes mellitus and overt nephropathy. Nephrol Dial Transplant.

[19] Bakris G, Burgess E, Weir M, Davidai G, Koval S (2008). Telmisartan is more effective than losartan in reducing proteinuria in patients with diabetic nephropathy. Kidney Int.

[20] Park JB, Sung K-C, Kang S-M, Cho EJ (2013). Safety and efficacy of fimasartan in patients with arterial hypertension (Safe-KanArb Study). Am J Cardiovasc Drugs.

[21] Eknoyan G, Lameire N, Eckardt K, Kasiske B, Wheeler D, Levin A (2013). KDIGO 2012 clinical practice guideline for the evaluation and management of chronic kidney disease. Kidney Int.

[22] Levey AS, Coresh J, Greene T, Stevens LA, Zhang YL, Hendriksen S (2006). Using standardized serum creatinine values in the modification of diet in renal disease study equation for estimating glomerular filtration rate. Ann Intern Med.

[23] Phillips A, Ebbutt A, France L, Morgan D (2000). The international conference on harmonization guideline “statistical principles for clinical trials”: issues in applying the guideline in practice. Drug Inform J.

[24] Kim TW, Yoo BW, Lee JK, Kim JH, Lee K-T, Chi YH (2012). Synthesis and antihypertensive activity of pyrimidin-4 (3H)-one derivatives as losartan analogue for new angiotensin II receptor type 1 (AT 1) antagonists. Bioorg Med Chem Lett.

[25] Kellici TF, Tzakos AG, Mavromoustakos T (2015). Rational drug design and synthesis of molecules targeting the angiotensin II type 1 and type 2 receptors. Molecules.

[26] Yi S, Kim T-E, Yoon SH, Cho J-Y, Shin S-G, Jang I-J (2011). Pharmacokinetic interaction of fimasartan, a new angiotensin II receptor antagonist, with amlodipine in healthy volunteers. J Cardiovasc Pharmacol.

[27] Klahr S, Levey AS, Beck GJ, Caggiula AW, Hunsicker L, Kusek JW (1994). The effects of dietary protein restriction and blood-pressure control on the progression of chronic renal disease. N Engl J Med.

[28] Wright JT, Bakris G, Greene T, Agodoa LY, Appel LJ, Charleston J (2002). Effect of blood pressure lowering and antihypertensive drug class on progression of hypertensive kidney disease: results from the AASK trial. JAMA.

[29] Appel L, Wright J, Greene T, Agodoa L, Astor B, Bakris G (2010). The long-term effects of a lower blood pressure goal on progression of hypertensive chronic kidney disease in African-Americans. New Engl J Med..

[30] Group AS (2010). Effects of intensive blood-pressure control in type 2 diabetes mellitus. N Engl J Med.

[31] Wright JT, Williamson JD, Whelton PK, Snyder JK, Sink KM, SPRINT Research Group (2015). A randomized trial of intensive versus standard blood-pressure control. N Engl J Med.

